# A mixed-reality stimulator for lumbar puncture training: a pilot study

**DOI:** 10.1186/s12909-023-04173-9

**Published:** 2023-03-22

**Authors:** Xiaojing Huang, Zhaoxia Yan, Chao Gong, Zheliang Zhou, Hua Xu, Chunhui Qin, Zhenwei Wang

**Affiliations:** 1 Department of Anesthesiology and Pain Medicine, Shanghai General Hospital, Shanghai Jiao Tong University School of Medicine, Shanghai, 200080 China; 2 Department of Anesthesiology, Yueyang Hospital of Integrated Traditional Chinese and Western Medicine, Shanghai University of Traditional Chinese Medicine, No. 110, Gan He Road, Hongkou District, Shanghai, 200437 China; 3 Department of Anesthesiology, Shanghai General Hospital, Shanghai Jiao Tong University School of Medicine, Shanghai, 200080 China; 4 Department of Respiration Medicine, Yueyang Hospital of Integrated Traditional Chinese and Western Medicine, Shanghai University of Traditional Chinese Medicine, No. 110, Gan He Road, Hongkou District, Shanghai, 200437 China

**Keywords:** Mixed reality, Stimulator, Lumbar puncture, Training

## Abstract

**Background:**

The simulation is one of the basic methods of medical education, which is often used for procedural skills training. However, the existing simulator lacks internal anatomical landmarks. The study developed a mixed-reality stimulator and evaluated its usability and feasibility in lumbar puncture training.

**Methods:**

The study recruited 40 subjects, including medical students, residents and faulty with varied levels of experience. Before training, participants completed the questionnaire about the basic information and watched a presentation about mixed reality. After practicing on mixed-reality stimulator, which provided internal anatomical structure, the examination was carried out and the results were documented. At the end of the training, trainees completed a survey of MR technology.

**Results:**

In this study, participants generally believed that the MR technology was very realistic (90%), and that the presentation of internal anatomy could help the operation (95%). Moreover, 72.5% and 75%, respectively, strongly agreed that the MR technology promoted learning and should be used in medical training. After this training, the success rate of puncture and the puncture time were significantly improved in experienced and non-experienced participants.

**Conclusion:**

The existing simulator was easy to be transformed into MR simulator. This study showed the usability and feasibility of MR simulator in lumbar puncture training. As a potentially good tool to simulated medical skills training, next, MR technology would be developed and evaluated in more clinical skills teaching scenarios.

## Background

Simulators are becoming increasingly important in medical education and its application in the field of anesthesiology and critical care medicine is currently being developed [1, 2]. The anesthetic and care medicine environment are challenging ones, which require a large number of precise skill procedures to complete the treatment. The repeated practice is exactly the approach encouraged by training via the simulator [3, 4].

However, traditional simulators often lack important internal landmarks and anatomical structures that can be identified by trainees. The information which displays is not intuitive and vivid enough to attract the attention of students and brings little gains to students in a specified time [5]. Virtual Reality (VR) and Augmented Reality (AR) have achieved good results in medical education, which can bring students an immersive feeling and a desire to learn actively [5, 6]. Especially in recent years when AR is integrated into anatomy education, it has been proved that AR improves students' understanding of anatomy relationship, spatial positioning, visual spatial ability, cognitive load, task time and academic performance [7, 8]. Unfortunately, they also have inevitable shortcomings. For example, the exclusion between virtual and real world and the lack of interaction [9].

Mixed reality (MR) technology has the characteristics of combination of virtual and reality, real-time interaction and accurate matching, and MR can combine virtual images with entities and display the internal structure of objects [10, 11]. As a technology with the potential to enhance anatomical education, MR can improve anatomical education by providing learners with a more immersive and interactive experience and also offer a more cost-effective alternative to traditional anatomical education methods, such as the use of MR to visualize and explore complex anatomical structures, to simulate surgical procedures, and to provide hands-on experiences for learners. These applications can help to improve student engagement, motivation, and retention of anatomical knowledge. However, MR technology is still in its early stages, and there are several challenges including the need for more robust and user-friendly hardware and software, as well as the development of more effective educational content [12].

Lumbar puncture is an essential clinical skill for the clinician, especially for anesthesiologists [13]. It is applied for spinal anesthesia or the intrathecal administration of drugs. The procedure also is of high importance for the diagnosis of some neurological diseases. However, Clinicians and students do not have enough lumbar puncture experience because of ethical problems, doctor-patient relationship, lack of practice and lack of self-confidence [13, 14].

In this study, 3D anatomy image matching the mannequin was developed by using MR technology based on HoloLens glasses. The mannequin showing the internal anatomical structure was applied to the practice of lumbar puncture. The aim of this study is to evaluate the usability and feasibility of the application of MR technology in the training of lumbar puncture.

## Method

This project was deemed exempt by Institutional Review Board of Shanghai Yueyang Integrated Traditional Chinese Medicine and Western Medicine Hospital affiliated to Shanghai University of Traditional Chinese Medicine.

**Fig. 1:**
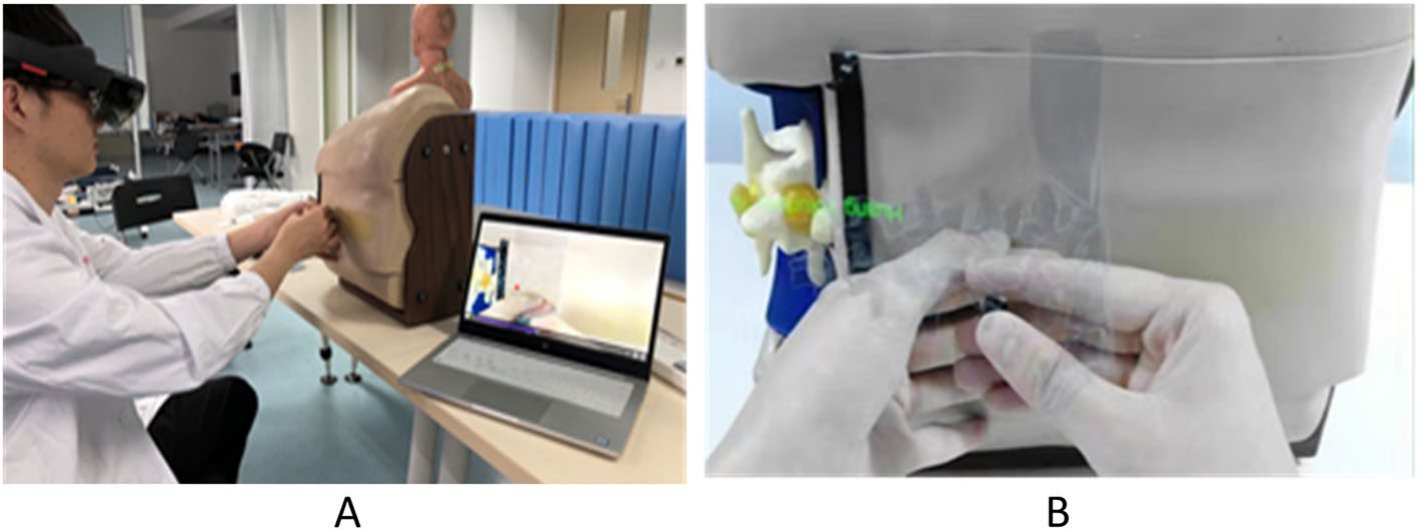
**A** The mixed reality simulator of lumbar puncture used in the study. In the virtual environment, the puncture process of the subjects was displayed on in real-time the laptop screen; **B** The 3-dimensional images were sighted with HoloLens smart glasses of Microsoft. The photo was taken with the camera of Glasses, was 2-dimensional and looked deformed compared to the 3-dimensional image seen in Glasses. In fact, in the glasses, it was a realistic 3D image

**Fig. 2:**
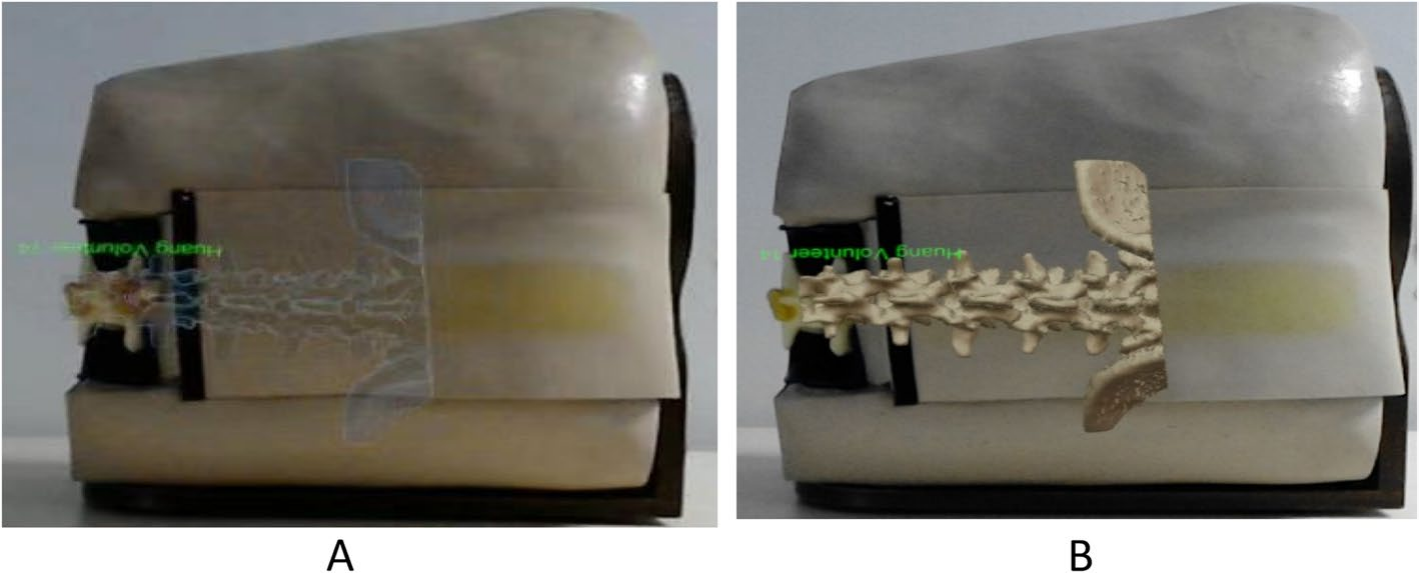
**A** Mixed reality virtual image of translucent material; **B** Mixed reality virtual image of bone material

The lumbar vertebrae were scanned by CT with a thin layer of 0.625 mm, and the images were saved and converted into 3D images using Medical Rapid Prototyping technology of Medical Mixed reality surgery navigation System. The 3D image models were scaled on the stimulator in a ratio of 1:1, and the important internal landmarks in stimulator and anatomical structures of stimulator were seen by trainees wearing HoloLens glasses at the same time (Fig. 1A). The 3-dimensional images were sighted with HoloLens smart glasses of Microsoft (Fig. 1B). The 3D images were presented in either translucent material or bone material (Fig. 2). The glasses used in this study was the first generation of HoloLens smart glasses of Microsoft. The Mixed reality technology used in this study was independently developed by Dr. Qin (Patent application No. CN201710414454 in China, 2019). Prior to the use of the simulator, two anesthesiologists who were not involved in the research and development evaluated the MR simulator based on their years of experience in anatomy and lumbar puncture, and judged it to be anatomically correct.

Participant included interns in anesthesiology, residents, and other inexperienced doctors who needed spinal puncture technique. Before the operation, participants completed a pre-study questionnaire to provide demographic data, including training level, experience and self-assessment confidence in the ability of lumbar puncture.

A total of 40 trainees were recruited in this study. Prior to the training, the trainees were assessed for lumbar puncture (LP) techniques and their scores were recorded. And then, participants received the same basic anatomy training of LP, provided by XH and CG. After that, participants were guided to use the MR stimulator, which was demonstrated by CQ and HX, including adjusting and testing glasses to ensure comfort and accuracy of presentation. In the meantime, participants strengthened their understanding of spine anatomy through Glasses following the advice given by the trainer.

The simulator used in the research was a common simulator without internal landmarks for lumbar puncture. Participant were asked to locate the L3-L4 interspace by palpation. The L3 and L4 spinous processes was correctly recognized, and the midpoint between the two spinous processes was used as the target of needle insertion. After the puncture space was accurately identified, the internal 3D image was projected on the simulator through the glasses, and the simulator with 3D image was presented on the computer screen at the same time. The trainees were allowed to understand the puncture position relative to the previously determined anatomical position. The trainees were asked to practice needle inserting with anatomy in view through glasses. Once participants were confident with their skill, they were asked to insert a needle on simulator without anatomy in view. If the needle inserted correctly into the subarachnoid space, the fluid flew out of the needle, simulating the outflow of cerebrospinal fluid. Otherwise, no liquid will flow out of the needle.

Through the evaluation of the questionnaire and tests, two experienced evaluators independently recorded trainees’ achievements. The main items of the questionnaire included the correct identification of the anatomy, the correct angle and direction of the puncture needle, and the number of the puncture attempts. The operation time from the beginning of locating the puncture point to the end of needle fixation and the fluid flowing out of needle without anatomy in view by glasses were also recorded. At the end of training, participants completed a post-study questionnaire to conduct for the feedback survey after training. A 0-10point Likert scale was used to assess MR simulator subjectively. The scores of each attempt were compared to study the potential relationship before and after training.

This was a new tool in teaching study for testing of usability and feasibility, so there were no articles to refer to, and no power analysis size was required. SPSS, Windows® version 23.0 (SPSS, Chicago, IL, USA), was used for data analysis. Continuous variables were expressed as Mean ± SD, and categorical variables as counts (percentages, %). The difference comparison of continuous variables was completed by Student’s t-test, while chi-square test was used to analyze categorical variables. A two-sided *P* value < 0.05 was considered statistically significant.

## Results

The demographics of 40 participants with varied levels of experiences, including students, intern, faulty, resident, nurse anesthetist and fellow, are described in Table 1. Prior experience and training with lumbar puncture and training were shown in Table 2. Only 2 of the 40 participants were trained based on simulator in lumbar puncture, 62.5% of participants had no experience with lumbar puncture, and two medical students had prior experience with lumbar puncture.

**Table 1:** Participant demographics

Specialty	n (%), *N* = 40
MS-4(8)/MS-5(10)	18(45.0)
Faculty	6(15.0)
Intern (PGY-1 – PGY-3)	6(15.0)
Resident (PGY-3 – PGY-5)	5(12.5)
Nurse Anesthetist	3(7.5)
Fellow	2(5)

*MS* Medical student, *PGY* Postgraduate year

**Table 2:** Experience with LP training and puncture

Experience	n/N (%)
Prior LP training	18/40 (45)
The training with simulator	2/18 (33.3)
The type of simulator-mannequin	2/2 (100)
Prior LP punctures:	
None	25/40 (62.5)
1–5	3/40 (7.5)
6–10	1/40 (2.5)
11–20	4/40 (10)
> 20	7/40 (17.5)

Table 3 showed results of the questionnaire survey, participants with and without prior LP training reported subjectively that the MR technology was very realistic (strongly agree 82.5%), easy to use (strongly agree, 75%), and very helpful to the training. It was reported that the MR technology helped to identify landmarks (strongly agree, 87.5%); was easier to learn internal anatomy (95%); made training interesting (82.5%); improved the confidence in LP skills (92.5); helped to improve the LP skills (72.5%); will be useful in the medical training (92.5%); was a useful tool of medical training (92.5%); was agreed to be applied in medical training (82.5%). MR technology’s novel features and functionalities, as well as the interaction between simulators and trainees, will promote learning (strongly agree, 87.5% vs 80.0%, respectively).

**Table 3:** Participant post-study evaluation. n (%), *N* = 40

Survey Item	0	1–3	4–7	8–10
MR anatomy was realistic	1(2.5)	2(5.0)	4(10)	33(82.5)
MR helped identify landmarks	3(7.5)	1(2.5)	1(2.5)	35(87.5)
Easier to learn internal anatomy	2(5.0)	0(0)	0(0)	38(95)
Easy to use	4(10)	1(2.5)	5(12.5)	30(75)
MR made training interesting	2(5.0)	3(7.5)	2(5.0)	33(82.5)
MR improved my confidence in LP skills	0(0)	0(0)	3(7.5)	37(92.5)
MR helped me to improve my LP skills	1(2.5)	3(7.5)	7(17.5)	29(72.5)
MR will be useful in my medical training	0(0)	2(5.0)	1(2.5)	37(92.5)
MR will promote learning				
Novel features and functionalities	1(2.5)	2(5.0)	4(10)	33(82.5)
Interaction between stimulator and trainee	2(5.0)	1(2.5)	5(12.5)	32(80.0)
MR was a useful tool of medical training	0(0)	1(2.5)	2(5.0)	37(92.5)
Agreement to apply MR in medical training	2(5.0)	2(5.0)	3(7.5)	33(82.5)

A scale of 0–10 was set, with 0 representing disagreement, 1 representing agreement and 10 representing strong agreement. Participants scored the questions based on their level of agreement with the item

There were significant differences in correct identification of the anatomy, correct angle and direction of the puncture needle and the numbers of the puncture attempts before and after training (*p* < 0.05) (Table 4). Before the training, 2 trainees had an operation time of more than 8 min and 2 trainees had an operation time of 7 min. After the training, only 1 trainee had an operation time of 4 min, and most of them were in 1–2 min. There was a significant statistical difference in the operation time before and after the training (*p* < 0.05) (Table 4).

**Table 4:** Changes in subjects before and after training (x ± s)

	Before training	After training	*P* Value
Correct identification of the anatomy	12/40	38/40	0.0001
Correct angle and direction of the puncture needle	22/40	35/40	0.0001
The numbers of puncture attempts	3.10 ± 2.09	1.68 ± 0.94	0.0001
Operation time (min)	4.08 ± 1.96	2.07 ± 0.92	0.0001

## Discussion

For the teaching and research department of the teaching hospital, the mixed-reality simulator is easy to develop. Teaching centers with Glasses and simulators can be carried out under the guidance of patented technology. The results of this study demonstrated the usability and feasibility of the application of mixed-reality stimulator in lumbar puncture training. According to the feedback from different participants, mixed reality technology had great advantages in medical training, which can improve learning and skills. In addition, the skills of experienced and inexperienced participants had been improved after the application of the mixed reality technique, which proved that the mixed reality technique could provide useful training techniques for the various experienced participants.

Compared to the AR technology, MR technology showed a significant improvement in knowledge retention in enhancing students' understanding of anatomy and physiology, in addition, it was reported higher levels of engagement and satisfaction with the technology than AR [15]. MR allowed participants to interact with 3D models and holographic images, which can help them better visualize and understand complex anatomical structures and physiological processes. It also allowed for more immersive and engaging learning experiences that can increase student motivation and interest in the subject matter [12]. In this study, mixed reality can provide a realistic simulation of procedures and allow participants to practice and refine their skills in a safe and controlled environment.

The application of MR in medicine is advancing rapidly. There is evidence to support its beneficial impact in many aspects of clinical teaching and medical training, such as medical teaching, preoperative discussion and programming, intraoperative navigation and remote consultation [11]. For example, a study found that in subclavian vein puncture training, mixed reality techniques improved supraclavicular puncture of subclavian vein and enhanced learners' confidence in operation [16, 17]. Another study showed that mixed reality techniques for surgical skills training have been proved to be realistic and useful [18, 19]. Mixed reality blends the physical and digital worlds and produces new environments and visualizations, where objects in two worlds co-exist and interact in real time. Mixed reality does not occur in the physical or virtual world, but is a hybrid of reality and virtual reality [20, 21]. The simulator used in this study is a standard mannequin with external anatomical markers. According to the size of the simulator vertebrae, MR technology matches the corresponding 3D vertebral virtual image on mannequin, so that the trainees can see the real simulator and the internal virtual anatomy, and the relationship between them at the same time [22, 23]. In this study HoloLens guided the subjects to the correct point of needle insertion and reminded them to pay attention to the mutual position between the needle and the vertebrae. Consequently, MR provided the trainees with a complete internal and external experience. At the same time, in the process of operation, the watching teachers could provide real-time guidance according to the operation of the participants. Such results helped participants to become more intuitive, simple and effective in learning the operation. Therefore, mixed reality technology may have great advantages and wide application in medical teaching in the future.

In addition, the purpose of this study is to create an efficient training environment to teach and evaluate procedural skills at a relatively low cost, and a standard simulator was selected instead of a 3D print simulator owning to the high price of 3D models. Furthermore, 3D printing technology may be more suitable for personalized medicine than medical teaching because 3D model is not typical and representative in basic medical teaching [24]. Moreover, MR can be used for a variety of rendering, magnification, cutting and selective visualization of image observation points, and it can be well integrated with simulators in the learning environment.

Every year, more than a million patients received lumbar puncture, with a failure rate of puncture about 30% in adults [25] and 40% to 50% in children [26]. In the simulation training, after a series of practice, it showed that the success rate of lumbar puncture had been improved in the simulation-based evaluation and in the actual lumbar puncture operation [27]. The familiarity of the internal mark position and the visual operation in the training would help the trainees to understand the normal and pathological state of the body, as well as the relationship between the operating behavior and what happened in the body. MR technology helped students understand the complex anatomy of the body intuitively, so that students could get more effective training. In this study, the ability to accurately identify the anatomy, to insert the needle at the right angle and direction, and the rate of successful puncture have been greatly improved after the training. In addition, the total operating time was significantly reduced compared with that before training.

This study was an attempt of MR in procedure skills. It was very important to find the potential limitations of this technology in medical education, which is helpful to improve teaching. The main complaints focused on HoloLens glasses in the study. For example, there was a slight time delay in the appearance of the glasses; the helmet was too heavy, causing soreness in the shoulder and neck; it was not very friendly to trainees who wear glasses. Despite these problems, they did not affect the trainees' ability to correctly identify the landmark structure and puncture. The research team is developing the real-time positioning and guidance function of the puncture needle in the procedure, which would further understand the relationship between the puncture needle and the surrounding tissues, as well as what may happen in the body. This improvement may increase the cost of teaching. However, as with any new technology, the cost of MR development and application will be reduced over time. The application of MR technology in medical education should be evaluated to ensure its efficacy and cost–benefit.

## Conclusion

This study demonstrates the usability and feasibility of mixed reality technology as a training tool for lumbar puncture. MR technology will provide a more immersive and effective training environment to learner to study more complex operations and will have a profound impact on the medical hands-on education. Thus, mixed reality is a promising and valuable approach that can be applied in medical simulation-education curricula in future.

## Data Availability

The datasets used and analyzed during the current study are available from the corresponding author on reasonable request.

## Abbreviations

MR: Mixed Reality
VR: Virtual Reality
AR: Augmented Reality
LP: Lumbar Puncture
MS: Medical student
PGY: Postgraduate year
